# PTEN/Akt Signaling Controls Mitochondrial Respiratory Capacity through 4E-BP1

**DOI:** 10.1371/journal.pone.0045806

**Published:** 2012-09-26

**Authors:** Chong Kiat Goo, Hwee Ying Lim, Qin Shi Ho, Heng-Phon Too, Marie-Veronique Clement, Kim Ping Wong

**Affiliations:** 1 Department of Biochemistry, Yong Loo Lin School of Medicine, National University of Singapore, National University Health Systems, Singapore, Singapore; 2 NUS Graduate School for Integrative Sciences and Engineering, Kent Ridge, Singapore, Singapore; University Health Network, Canada

## Abstract

Akt, a serine/threonine kinase has been shown to stimulate glycolysis in cancer cells but its role in mitochondrial respiration is unknown. Using PTEN-knockout mouse embryonic fibroblasts (MEF^PTEN−/−^) with hyper-activated Akt as a cell model, we observed a higher respiratory capacity in MEF^PTEN−/−^ compared to the wildtype (MEF^WT^). The respiratory phenotype observed in MEF^PTEN−/−^ was reproduced in MEF^WT^ by gene silencing of PTEN which substantiated its role in regulating mitochondrial function. The increased activities of the respiratory complexes (RCs) I, III and IV were retained in the same relative proportions as those present in MEF^WT^, alluding to a possible co-ordinated regulation by PTEN/Akt. Using LY294002 (a PI3K inhibitor) and Akt inhibitor IV, we showed that the regulation of enzyme activities and protein expressions of the RCs was dependent on PI3K/Akt. There was insignificant difference in the protein expressions of mitochondrial transcription factor: peroxisome proliferator-activated receptor gamma coactivator 1-alpha (PGC-1α) and its downstream targets, the nuclear respiratory factor 1 (NRF-1) and mitochondrial transcription factor A (mtTFA) between MEF^PTEN−/−^ and MEF^WT^. Similarly, mRNA levels of the same subunits of the RCs detected in Western blots were not significantly different between MEF^PTEN−/−^ and MEF^WT^ suggesting that the regulation by Akt on mitochondrial function was probably not via gene transcription. On the other hand, a decrease of total 4E-BP1 with a higher expression of its phosphorylated form relative to total 4E-BP1 was found in MEF^PTEN−/−^, which inferred that the regulation of mitochondrial respiratory activities by Akt was in part through this protein translation pathway. Notably, gene silencing of 4E-BP1 up-regulated the protein expressions of all RCs and the action of 4E-BP1 appeared to be specific to these mitochondrial proteins. In conclusion, PTEN inactivation bestowed a bioenergetic advantage to the cells by up-regulating mitochondrial respiratory capacity through the 4E-BP1-mediated protein translation pathway.

## Introduction

PTEN (phosphatase and tensin homology deleted on chromosome 10) is one of the tumor suppressors frequently lost in cancers [1], [2]. It has both protein and lipid phosphatase activities; the latter is associated with tumor suppression [3], [4]. By dephosphorylating phosphatidylinositol-3,4,5-trisphosphate (PIP_3_), PTEN prevents the membrane recruitment and activation of Akt which promotes cell survival, proliferation, growth and glycolysis [5], [6]. The ability of Akt in stimulating cell proliferation is best illustrated in Cowden and other PTEN hamartoma tumor syndromes [7]. The benign nature of these tumors could be due to the fact that neither PTEN nor Akt alone is sufficient for the cell to become cancerous [8], [9]. The signaling downstream of Akt is integrated by mTOR (mammalian target of rapamycin) and transduced to ribosomal protein kinase S6 kinase (S6K) and the eukaryotic translation initiation factor 4E (eIF4E)-binding proteins (4E-BP1, 2 and 3) to regulate protein translation [10]. 4E-BP1 is a repressor of 5′cap-dependent mRNA translation and it is inactivated upon phosphorylation by mTORC1. The phosphorylated 4E-BP1 is then released from the eukaryotic initiation factor 4E (eIF4E) which subsequently binds eIF4G to begin protein translation [11]. 4E-BP1 is a key effector of oncogenic activation of the Akt and ERK signaling pathways that integrate their function in tumors [12]. It correlates with the clinical findings that expression of high level of phosphorylated 4E-BP1 is associated with poor prognosis in several types of tumor, independent of the alteration of upstream oncogenic signaling [13].

While the activation of Akt by defective respiration in cancer cells has been shown to stimulate aerobic glycolysis [14], [15], the action of Akt on the mitochondrial respiration in cell transformation remains largely unknown. In this study, MEF^WT^ and MEF^PTEN−/−^ were used as a model to examine if the PTEN/Akt pathway has an effect on oxidative metabolism in normal and transformed cells. We compared a number of mitochondrial parameters and found that the MEF^PTEN−/−^ with hyper-activated Akt exhibited higher enzyme activities and protein expressions of all respiratory complexes. Pre-treatment of MEF^PTEN−/−^ with LY294002 (LY), an inhibitor of PI3K and mTOR [16] and Akt inhibitor IV decreased (a) the phosphorylation of Akt at both Ser^473^ and Thr^308^, (b) the phosphorylation of 4E-BP1 at Thr^37/46^ and (c) the expressions of RC I, III and IV. We also measured the mRNAs of the same subunits of the RCs detected in Western blot**s** and the protein expressions of the peroxisome proliferator-activated receptor gamma coactivator 1-alpha (PGC-1α), the nuclear respiratory factor 1 (NRF-1) and mitochondrial transcription factor (mtFTA) and showed that the regulation of mitochondrial function by Akt was probably not dependent on transcription. In conclusion, the higher proliferation in MEF^PTEN−/−^ was accompanied by increased mitochondrial respiratory capacity. The protein expressions of the RCs in MEF^PTEN−/−^ are attenuated by LY294002 and Akt inhibitor IV but are increased in MEF^WT^ by siRNA targeting PTEN or 4E-BP1, suggesting a regulation of mitochondrial respiratory activities by the PTEN/Akt/mTOR pathway through 4E-BP1-mediated protein translation.

## Experimental Procedures

### Cell culture

The MEF^WT^ and MEF^PTEN−/−^ cell lines [6] were kindly provided by Dr. Tak W. Mak of University of Toronto. They were cultured in DMEM (Sigma, D1152) with 100 U/ml penicillin, 100 µg/ml streptomycin, 2 mM glutamine and 10% fetal bovine serum in 5% CO_2_ at 37°C.

### Respiratory flux

The measurement of respiratory flux in intact MEF cells in a high-resolution respiratory system (Oroboros, Oxygraph-2k) was carried out according to the protocol described [17]. Cellular routine (R) state was established with the presence of cells in 2 ml of culture medium in the chamber at 37°C with constant stirring for 15–30 min. In the absence of exogenous substrates, the R (routine) respiration is dependent on endogenous substrates and substrates present in the culture medium. Following the stabilization of R, oxygen consumption is measured in the presence of 2 µg/ml oligomycin to obtain the Leak (L) state. In this non-physiological state, L respiration reflects intrinsic uncoupling of the mitochondria. Subsequently, a maximal electron transport system capacity (E) of the mitochondria is achieved by titrating sequentially with repeated doses of 1 µl from 1 mM FCCP until a maximal response is achieved. The net Routine/E is then calculated by applying the formula: Net routine/E = (R−L)/E. This parameter therefore represents the net oxygen consumption coupled to ATP biosynthesis, taking into account the leak component.

### Isolation of mitochondria

The procedure described in [18] was used with slight modifications. Cells were trypsinized, centrifuged at 1000 *g* for 5 min and the pellet was re-suspended in 1 ml of medium containing 250 mM sucrose, 20 mM Hepes, 10 mM KCl, 1.5 mM MgCl_2_, 1 mM EDTA and 1 mM EGTA, pH 7.4. This was followed by homogenization with 40 up-and-down strokes using a hand-held glass pestle. The mixture was then centrifuged at 800 *g* for 10 min and the supernatant obtained was subjected to another step of centrifugation at 14,000 *g* for 15 min. The final pellet was re-suspended in the same medium and was used immediately for ATP biosynthesis and measurement of MMP or kept at −80°C as freeze-thawed mitochondrial extracts for other enzyme assays.

### ATP biosynthesis

ATP production in 5 min was measured from the oxidation of the following substrates ± inhibitor: 10 mM succinate+5 µM rotenone, 5 mM each malate/glutamate, or 5 mM ascorbate and 1 mM tetramethylphenylenediamine (TMPD)+2 µg/ml antimycin A. 125 µM ADP and 30 µg mitochondrial protein were used in the reaction which was stopped by placing the tubes in a heating block at 100°C for 3 min, followed by centrifugation at 14,000 *g* for 15 min. ATP formed was measured by the luciferin/luciferase reaction and the luminescence produced was read in a luminometer (Victor 3, Perkin-Elmer) as described previously [19], [20].

### Measurement of mitochondrial membrane potential (MMP)

The membrane potential was measured in isolated mitochondria using JC-1, a specific mitochondrial probe, by a change in the ratio of red fluorescence of the J-aggregates to that of the green fluorescence of its monomers at Ex/Em wavelengths of 485/595 nm and 485/535 nm, respectively. Routinely, 0.2 µM JC-1, 125 µM ADP and 0.5 mg of mitochondrial protein were used with the following added sequentially: 5 mM each glutamate/malate, 5 µM rotenone; 10 mM succinate and 10 mM malonate.

### Activities of tricarboxylic acid cycle (TCA) enzymes

Assay conditions for measuring citrate synthase (CS) malate dehydrogenase (MDH) and glutamate dehydrogenase (GDH) in the freezed-thawed mitochondrial extracts have been described previously [21].

### NADH dehydrogenase or RCI

The enzyme activity of RCI was measured by a decrease in fluorescence of NADH in 5 min at excitation wavelength 352 nm and emission wavelength 464 nm using decylubiquinone as an electron acceptor [22]. A mitochondrial extract containing 20 µg protein was added to a respiratory buffer of pH 7.2 containing 25 mM KH_2_PO_4_, 2 mM NaN_3_, 5 mM MgCl_2_, 64 µM decylubiquinone, 2 mg/ml albumin and 25 µM NADH.

### Succinate dehydrogense or RCII

The assay was based on a decrease in absorbance of 2,6-dichlorophenolindolephenol (DCPIP) at 600 nm monitored for 5 min, with decylubiquinone as an acceptor [23]. A mitochondrial extract containing 30 µg protein was employed in the presence of 1 µM rotenone, 2 µg/ml antimycin A and 2 mM NaN_3_ to inhibit RCI, III and IV, respectively. A pre-incubation with succinate was carried out at 37°C for 5 min with the mitochondrial extract to overcome the known inhibitory effect of oxaloacetate on RCII.

### Ubiquinol dehydrogenase or RCIII

This activity was measured by a coupled assay using reduced decylubiquinone and cytochrome c (cyt c) as described [24]. The assay was started by adding 50 µM cyt c to a buffer containing 250 mM sucrose, 1 mM EDTA and 50 mM Tris-HCl, pH 7.4 and 30 µg mitochondrial protein. The absorbance of reduced cyt c was followed for 5 min at 550 nm.

### Cytochrome c oxidase or RCIV

The assay measured the rate of decrease of reduced cyt c as reported previously [25]. Cyt c was first reduced by adding 4 mg ascorbate to 10 mg/ml cyt c. The reaction was started by adding 80 µg mitochondrial protein to a solution containing 4 mM KH_2_PO_4_ and reduced cyt c. The decrease in absorbance of reduced cyt c was followed at 550 nm for 20 sec.

### F_o_F_1_-ATPase or RCV

This was measured by coupling the ADP formed to pyruvate kinase using phosphoenolpyruvate as the substrate. Pyruvate formed was reduced to lactate by lactate dehydrogenase (LDH) as described previously [25], [26]. The decrease in NADH was monitored for 10 min at 340 nm in an absorbance microplate reader.

### Determination of mitochondrial protein

The protein concentrations of the mitochondrial fraction and the whole cell lysate prepared from MEF^WT^ and MEF^PTEN−/−^ were determined by the bicinchoninic acid assay [27]. The purity of the mitochondrial fraction was examined by Western blot analysis using the mitochondrial and cytosolic markers, voltage dependent anion channel (VDAC) and Cu-Zn superoxide dismutase (SOD-1), respectively.

### Western blot analysis

Aliquots of the mitochondrial or cytosolic fractions or total cell lysates containing 50 µg protein were subjected to electrophoresis on sodium dodecyl sulfate-polyacrylamide gel and electro-blotted onto a nitrocellulose membrane. After blocking with 10% non-fat milk in TBS with 0.1% Tween-20, the membranes were probed with the respective primary antibodies of PTEN, Akt, p-Ser^473^Akt, p-Thr^308^Akt, 4E-BP1, p-Thr^37/46^ 4E-BP1, B-cell lymphoma 2 (Bcl-2) (Cell Signaling Technology, Beverly, MA, USA), VDAC (Calbiochem, San Diego, CA, USA) malic enzyme 1 (ME 1), hexokinase II (HK II), pan-actin (product no. sc-1615), PGC-1 α, NRF-1, mtTFA, SOD-1 (Santa Cruz Biotechnology, CA, USA), lactate dehydrogenase isoenzyme V (LDH-V) (Abcam, Cambridge, UK), uncoupling protein 2 (UCP2) (Alpha Diagnostic, Texas, USA), α-tubulin (Sigma-aldrich, St. Louis, MO, USA) and aconitase 2 (ACO2) (Abgent, San Diego, CA USA). Western blot analysis of the respiratory complexes was carried out using a cocktail containing antibodies against the following subunits of the five RCs: complex I subunit NDU FB8, complex II subunit 30 kDa (SdhB), complex III subunit Core 2 (RC III core 2), complex IV subunit I (CO1) and ATP synthase α-subunit (ATP5 F_1_α1) (MitoSciences, Oregon, USA). All primary antibodies were used at a dilution of 1∶1000 except for α-tubulin and pan-actin, the loading controls, which were diluted 1∶20000. Anti-goat pan-actin was used as the loading control for the Western blot analysis of the respiratory complexes because the α-subunit of ATP synthase and α-tubulin of same species (mouse) have similar molecular weight and therefore could not be resolved.

### Inhibition by LY294002 and Akt inhibitor IV

Briefly, MEF^PTEN−/−^ grown to 70% confluence were pre-incubated with either 25 µM LY for 4 h or 1 µM Akt inhibitor IV for 8 h prior to enzymatic assays and Western blot analyses.

### RNA extraction and reverse transcription

Total RNA from MEF^WT^ and MEF^PTEN−/−^ was isolated using TRIzol reagent (Invitrogen, Carlsbad, CA, USA) according to the manufacturer's instructions. 500 ng RNA was reverse transcribed using 1 µl of ImPromII reverse transcriptase and 0.5 µg random hexamer for 60 min at 42°C and terminated at 70°C for 5 min according to the instruction from the manufacturer (Promega, Madison, WI, USA).

### Primers and real-time PCR

The mRNAs of the same five RC subunits detected in Western blot analyses were quantified by real-time PCR by using their respective forward and reverse primers as shown in Table S1. The mRNA of 4E-BP1 was detected using forward primer: 5′-GGACCAGCCGTAGGAC-3′, reverse primer: 5′-TGAGTGAGGAGCAGGAC-3′. Mouse alpha 1A tubulin (forward primer: 5′-TCGATGAAGTTCGCACCG-3′, 5′-reverse primer: TATTGGCAGCATCCTCCT-3′) was used as an internal control for normalization. Real time PCR using SYBR Green I was performed on the iCycler iQ (Biorad, Hercules, CA, USA) in a total volume of 25 µl containing 1× Xtensa-Mix-SG (BioWORKS, Singapore), 1 µl of cDNA sample, 2.5 mM MgCl**_2_**, 0.2 µM of each primer, and 0.5 U KlearTaq DNA polymerase (KBiosciences, Hertfordshire, UK). Real-time PCR was carried out after an initial denaturation of 10 min at 95°C followed by 40 cycles of 20 s denaturation at 95°C, 20 s annealing at 60°C and 20 s extension at 72°C. Detection of fluorescence was carried out at the annealing phase. The threshold cycles (Ct) were calculated automatically using the iQ5 software (Bio-Rad).

### SiRNA targeting PTEN and 4E-BP1

Gene silencing of PTEN on MEF^WT^ and of 4E-BP1 on both MEF^WT^ and MEF^PTEN−/−^ were carried out using small interference RNAs. Cells at 60–70% confluence were transfected for 16 h with either (a) 20 nM PTEN (sense: CGAUCUUGACAAAGCAAACtt, antisense: GUUUGCUUUGUCAAGAUCGtt purchased from Ambion, NY) or (b) with 4E-BP1-targeting RNA which consists of a pool of 3 target-specific 4E-BP1 siRNAs (product no. sc-29595 from Santa Cruz Biotechnology, CA, USA) or (c) with non-targeting RNAs r(UUCUCCGAACGUGUCACGU)d(TT) and r(ACGUGACACGUUC GGAGAA)d(TT) from Qiagen, Hilden, Germany. Following 18 h of recovery in normal culture medium, the efficiency of knockdown of PTEN and 4E-BP1, the effects of PTEN and 4E-BP1 knockdown on RC expressions and the effect of PTEN knockdown on respiratory flux were measured.

### Statistical Analysis

Data were presented as means ± SD, and were analyzed by the Student's t-test where a *p* value of <0.05 was considered significant.

## Results

### Characterization of MEF ^PTEN−/−^

**Figure 1:**
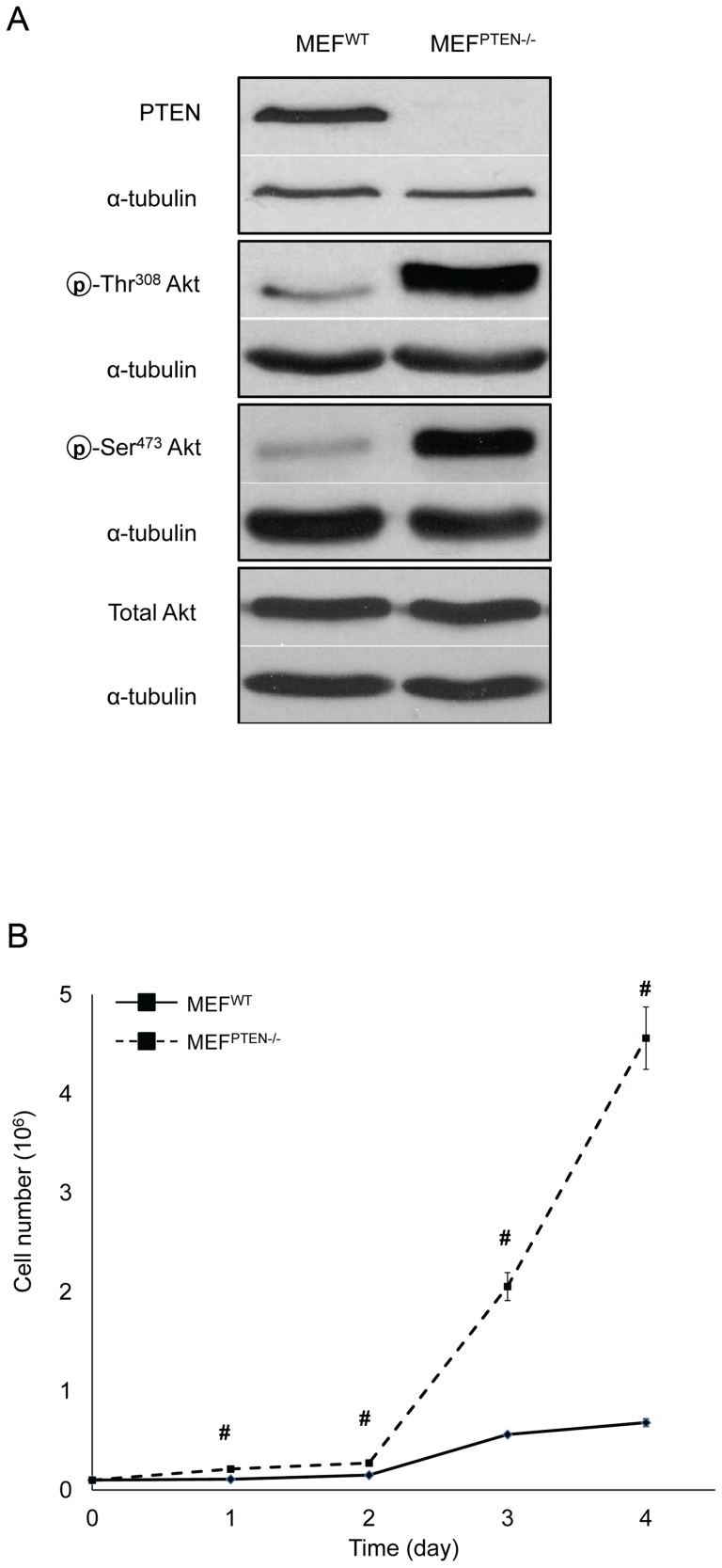
Characterization of MEF^PTEN−/−^. (**A**) *Western blot analyses of PTEN, Akt, p-Ser^473^Akt and p-Thr^308^Akt* Immunoblotting of PTEN, Akt, p-Ser^473^Akt and p-Thr^308^Akt was carried out on whole cell lysates of MEF^WT^ and MEF^PTEN−/−^. The loss of PTEN in MEF^PTEN−/−^ resulted in hyperactivation of Akt on both phosphorylation sites without affecting the expression of total Akt. The respective loading controls of α-tubulin were included. (**B**) *Rate of cell proliferation* This was measured by the trypan-blue exclusion assay. The MEF^PTEN−/−^ has a significantly higher rate of proliferation compared to MEF^WT^ from days 1–4 of culture. # *p*<0.005 for n = 3.

We first confirmed the PTEN knockout phenotype in MEF^PTEN−/−^ by immunoblotting the protein expressions of PTEN, total and phospho-Akts in the whole cell lysate. The absence of PTEN causing Akt hyperactivation was evident by the higher expressions of p-Ser^473^Akt and p-Thr^308^Akt without a significant change in the total Akt (Fig. 1A). In the absence of PTEN, a significantly higher rate of proliferation was observed in MEF^PTEN−/−^ from day 1 to 4 (Fig. 1B) with a doubling time of 22 hr and 16 hr, respectively, for MEF^WT^ and MEF^PTEN−/−^ (data not shown).

### Oxidative phenotype in MEF ^PTEN−/−^

#### ATP biosynthesis

**Figure 2:**
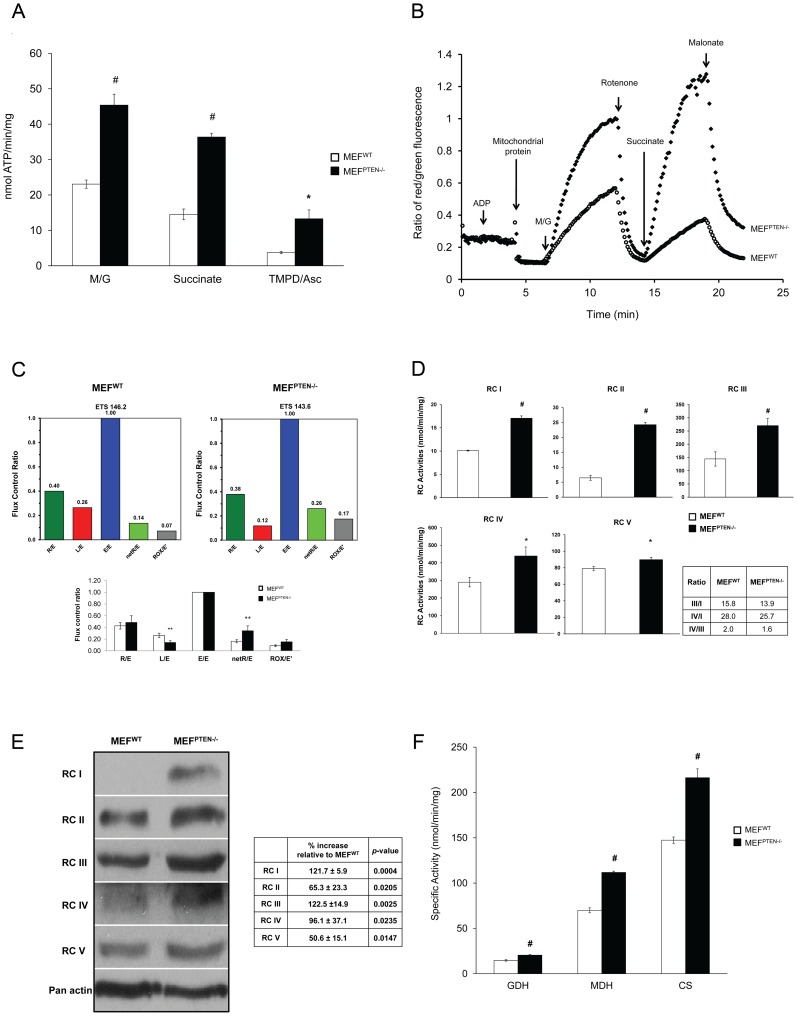
Mitochondrial parameters in MEF^WT^ and MEF^PTEN−/−^. (**A**) *Biosynthesis of ATP* This was carried out in isolated mitochondria which showed a higher rate of ATP generation in MEF^PTEN−/−^ compared to MEF^WT^. The respiratory substrates (± inhibitor) were 5 mM each of malate/glutamate (M/G), 10 mM succinate+5 µM rotenone, and 1 mM TMPD and 5 mM ascorbate in the presence of 2 µg/ml antimycin A. * *p*<0.05, # *p*<0.005 for n = 3. (**B**) *Mitochondrial membrane potential* The cationic dye, JC-1was used to monitor the mitochondrial membrane potential (MMP). JC-1 monomers (green fluorescence) form J-aggregates (red fluorescence) in the matrix upon energization by added respiratory substrates namely malate/glutamate (M/G, 5 mM each) and succinate (10 mM). Rotenone (5 µM) and malonate (10 mM), inhibitors of RCI and II, respectively, decreased the MMP as reflected by an attenuation of the ratio of red∶green fluorescence. Mitochondrial protein used was 0.5 mg. Data presented is representative of 3 independent experiments. (**C**) *Oxygen consumption* The NetR/E (where R = Routine and E = maximal electron transport system capacity) was obtained as described under “Experimental procedures” using an Oxygraph 2k (Oroboros). A higher NetR/E was observed in MEF^PTEN−/−^ compared to MEF^WT^ as shown in a representative set of data. On the other hand, Leak/E was always lower in MEF^PTEN−/−^. A significant difference in NetR/E between the two cell lines showed that the electron transport system was more efficient in driving ATP synthesis in MEF^PTEN−/−^ compared to MEF^WT^ as shown in Fig. 2C inset; the latter was based on three independent sets of experiments with ** *p*<0.01 for n = 3. (**D**) *Enzyme activities of the RCs* These were expressed in nmol/min/mg protein. They were measured as described in the “Experimental procedures”. Ratios of RC III/I, IV/I and IV/III are shown in the inset. * *p*<0.05, # *p*<0.005 for n = 3. (**E**) *Western blot analyses of respiratory complexes* Whole cell lysates of MEF^WT^ and MEF^PTEN−/−^were probed with a cocktail containing antibodies against various subunits of the five RCs as described in the text. Quantification of the bands by the ImageJ software showed % increase in protein expressions of RCs in MEF^PTEN−/−^ relative to MEF^WT^. A higher degree of increase in RCI, III and IV, as compared to RCII and V was apparent (Fig. 2E inset). (**F**) *Activities of TCA cycle enzymes* Glutamate dehydrogenase (GDH), malate dehydrogenase (MDH) and citrate synthase (CS) were measured in freeze-thawed mitochondrial extracts as described in [21]. # *p*<0.005. for n = 3.

The overall function of mitochondria in energy production was measured by the rate of ATP biosynthesis in isolated intact mitochondria upon the addition of respiratory substrates. As shown in Fig. 2A, the rate of ATP biosynthesis from the oxidation of malate/glutamate for RCI, succinate for RCII and TMPD/ascorbate for RCIV was significantly higher in MEF^PTEN−/−^ compared to the MEF^WT^.

#### Mitochondrial Membrane potential (MMP)

The generation of mitochondrial membrane potential is often used as an indicator to assess mitochondrial respiratory function. We measured the MMP of intact mitochondria using the JC-1 dye with serial addition of substrates and inhibitors. The uptake of JC-1, a cationic dye, depends essentially on the MMP. The oxidation of respiratory substrates such as malate/glutamate and succinate added to mitochondria induced the generation MMP, resulting in an increased entry of JC-1 monomers which exhibited a green fluorescence. With enhanced accumulation, the monomers combined to form J-aggregates which showed a red fluorescence. The ratio of red∶green fluorescence measured the magnitude of the MMP. The MMP induced by malate/glutamate and succinate was higher in the MEF^PTEN−/−^ compared to MEF^WT^ as shown in Fig. 2B. Inhibition of RCI by rotenone and of RCII by malonate confirmed the measurement and functionality of RCI and RCII.

#### Respiratory Flux

To investigate the effect of PTEN/Akt on mitochondrial function, oxygen consumption was measured with intact cells in normal culture medium. As compared to isolated mitochondria, the intact cell analysis allowed an integration of regulatory events by which cytoplasmic and nuclear factors influenced mitochondrial function. Net R/E (where R = Routine and E = ETS) combined basal oxygen consumption (Routine), oligomycin-inhibited respiration (Leak) and maximal capacity of electron transport system (E) to provide a parameter R/E which measured the proportion of respiratory capacity coupled to oxidative phosphorylation. As shown in a representative set of data for MEF^PTEN−/−^ and MEF^WT^ (Fig. 2C), the former had higher Net Routine/E, which inferred that a higher proportion of oxygen consumption of the electron transport system was coupled to ATP synthesis. This could be contributed by a lower degree of leak respiration, expressed as Leak/E as shown in Fig. 2C inset, which was obtained from three independent experiments.

#### Activities and protein expressions of respiratory complexes

The higher MMP and rate of ATP biosynthesis in MEF^PTEN−/−^ observed above suggested a possible increase in the electron transport system coupled to the phosphorylation of ADP. This was reflected in the data of respiratory flux above. Indeed, extracts of mitochondria isolated from MEF^PTEN−/−^ showed higher activities of all five respiratory complexes compared to the MEF^WT^ (Fig. 2D). Interestingly, the ratios of RC III/I, IV/I, and IV/III in the MEF^PTEN−/−^ were similar in magnitude to those in the MEF^WT^ (Fig. 2D, inset). This observation alluded to a coordinated regulation of these RCs via transcription, translation and/or post-translation. At the protein level, we observed higher expressions of all five RCs in the MEF^PTEN−/−^ compared to the MEF^WT^ (Fig. 2E). Quantification of the bands using the ImageJ software showed a significant difference based on triplicate measurements and a higher magnitude of increase in RCI, III and IV compared to RCII and V (Fig. 2E inset).

#### Tricarboxylic acid (TCA) cycle enzymes

To sustain a higher MMP and rate of ATP biosynthesis, more reducing equivalents must be fed into the electron transport system from the TCA cycle. We found the activities of glutamate dehydrogenase (GDH) and malate dehydrogenase (MDH), two NAD-linked enzymes as well as citrate synthase (CS), a mitochondrial marker enzyme, were significantly higher in the MEF^PTEN−/−^ as compared to the MEF^WT^ (Fig. 2F).

### Enhanced mitochondrial function in MEF^PTEN−/−^

#### (a) Akt hyperactivation

**Figure 3:**
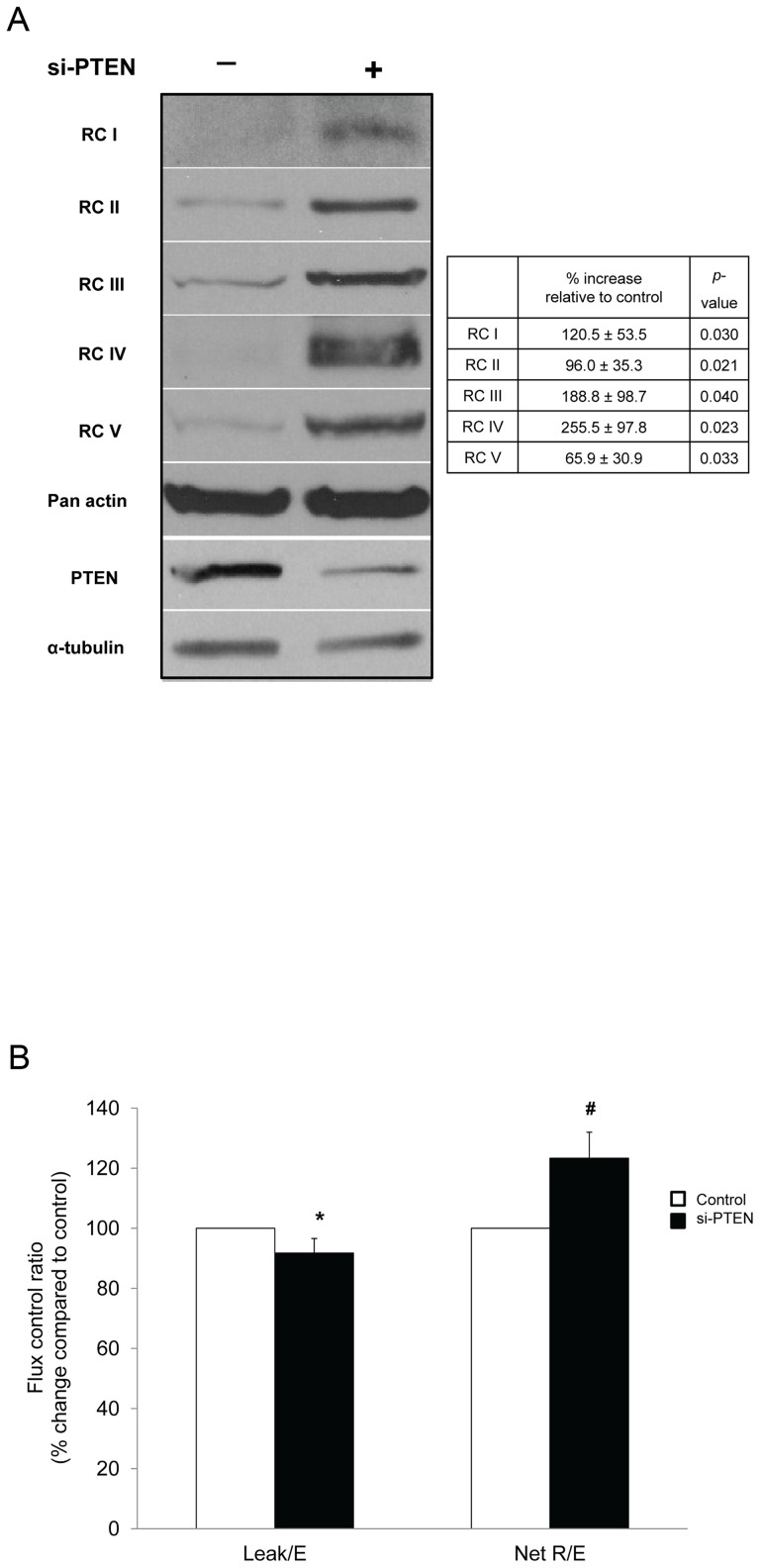
RNA-silencing PTEN in MEF^WT^. (**A**) *Western blot analyses of respiratory complexes* The protein expressions of all five RCs were significantly up-regulated by si-RNA of PTEN. A higher degree of increase in RCI, III and IV, as compared to RCII and V was apparent (inset). (**B**) *Oxygen consumption* si-RNA of PTEN was accompanied by an increase in the net oxygen consumption expressed as NetR/E and a decreased Leak/E when compared to untreated MEF^WT^. * p<0.05, # p<0.005 for n = 3.

**Figure 4:**
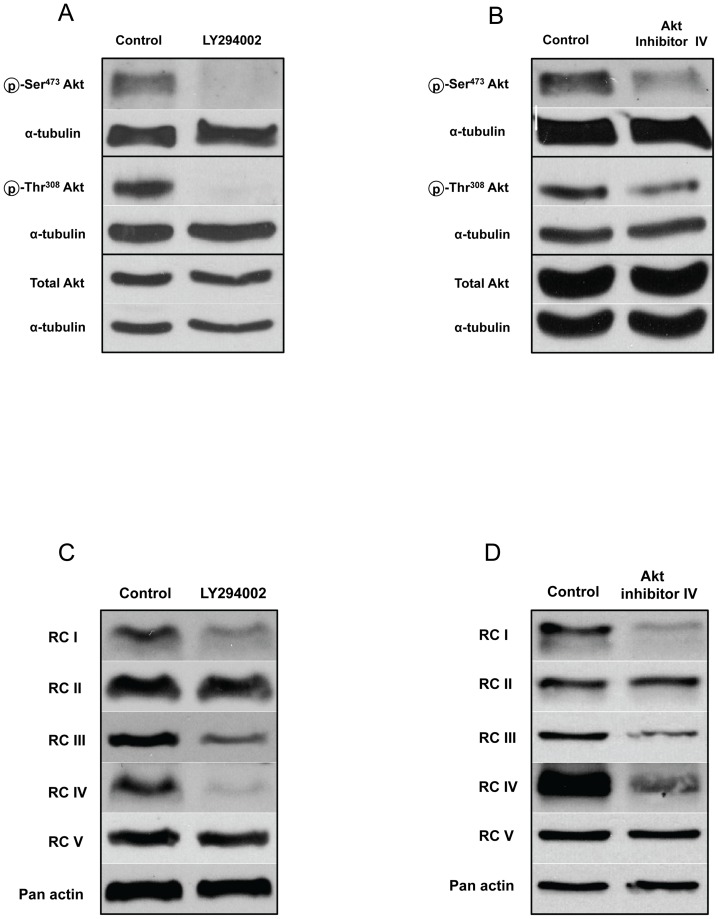
Effects of LY294002 and Akt inhibitor IV on MEF^PTEN−/−^. MEF^PTEN−/−^ were exposed to 25 µM LY294002 for 4 h or 1 µM Akt inhibitor IV for 8 h. (**A, B**) *Western blot analyses of p-Ser^473^Akt, p-Thr^308^Akt and total Akt* Expressions of p- Ser^473^Akt, p-Thr^308^Akt were compromised by LY and Akt inhibitor IV, respectively, while total Akt remained relatively unchanged. (**C, D**) *Western blots of respiratory complexes* Protein expressions of respiratory complexes I, III and IV were down regulated by LY and Akt inhibitor IV. Those of RCII and RCV appeared to be unaffected.

A significantly higher protein expression of all RCs was found in MEF^WT^ with siRNA knockdown of PTEN (Fig. 3A and inset). The PTEN-knockdown cells exhibited higher Net R/E with lower Leak/E (Fig. 3B), suggesting that PTEN aberration could be responsible for the enhanced coupling between oxidation and phosphorylation in mitochondria isolated from MEF^PTEN−/−^ (shown previously as NetR/E in Fig. 2C). To further explore if the higher mitochondrial respiration in MEF^PTEN−/−^ was attributed to hyperactivated Akt, inhibition of the PI3K/Akt signaling pathway was attempted with LY294002, known to act on PI3K and mTOR [16], [28], [29] and Akt Inhibitor IV, an ATP-competitive inhibitor of Akt autokinase activity [30]. Both inhibitors reduced the phosphorylation at Ser^473^ and Thr^308^ of Akt without affecting the total Akt (Fig. 4A and 4B). The reduction in Akt phosphorylation by these two inhibitors correlated with (a) decreased enzyme activities of all four electron-transport complexes (I to IV) in MEF^PTEN−/−^ (Table 1) and (b) repression of the protein expressions of RCI, III, and IV (these are involved in proton pumping) in MEF^PTEN−/−^ (Fig. 4C and 4D). The protein expressions of RCII and V were not or minimally affected.

**Table 1:** Inhibition of enzyme activities of respiratory complexes.

	% inhibition	
	LY294002	Akt inhibitor IV
**RCI**	14.9±0.87#	66.5±6.99#
**RCII**	33.7±9.18**	38.6±7.89**
**RCIII**	26.8±8.25**	38.1±1.01#
**RCIV**	21.3±4.41**	42.9±6.06#

Activities of respiratory complexes I to IV were inhibited by LY294002 (inhibitor of PI3K and mTOR) and Akt inhibitor IV. Values are expressed as % inhibition compared to untreated controls.

** p<0.01 and

# p<0.005 for n = 3.

#### (b) Regulation of gene transcription

**Figure 5:**
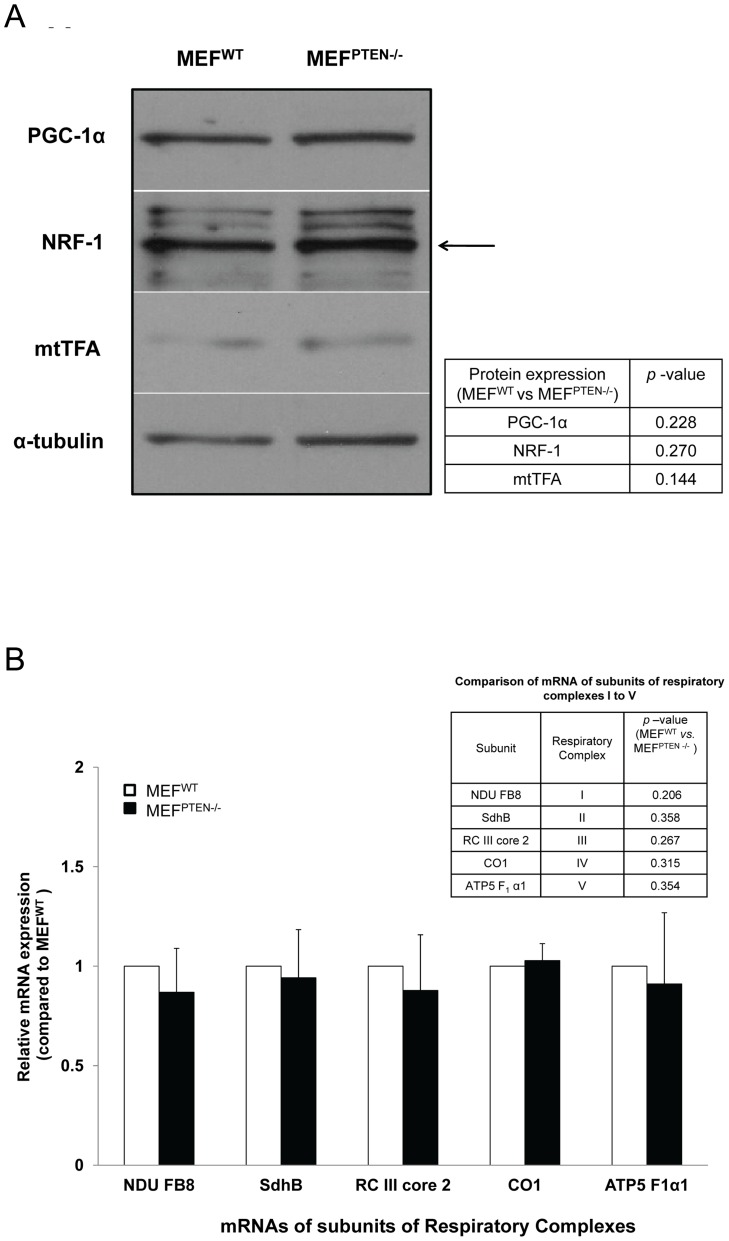
Transcriptional Regulation. (**A**) *Western blots of mitochondrial transcription factors* The protein expressions of peroxisome proliferator-activated receptor gamma coactivator 1-alpha (PGC-1α), nuclear respiratory factor-1 (NRF-1) and mitochondrial DNA transcription factor A (mtTFA) were measured in MEF^WT^ and MEF^PTEN−/−^. Quantitative analysis showed no significant difference between the two (inset). (**B**) *Measurement of mRNA by real-time PCR* The mRNAs of the five subunits RCs used in our Western blot analysis were not significantly different in MEF^PTEN−/−^ and MEF^WT^ with *p* values shown in the accompanying table (inset). Mouse alpha 1A tubulin was used for normalization.

The peroxisomal proliferator activated receptor γ coactivator-1α (PGC-1α) and its downstream targets: nuclear respiratory factor-1 (NRF-1) and mitochondrial DNA transcription factor A (mtTFA) are known to enhance respiratory function by acting on several nuclear genes encoding subunits of the respiratory complexes [31], [32]. The level of these proteins could provide some indication if transcriptional regulation played a role in the Akt-mediated up-regulation of mitochondrial activities observed in MEF^PTEN−/−^. No significant difference was observed in their protein expressions (Fig. 5A and inset). Similarly, the mRNA levels of the five subunits of respiratory RCs used in our Western blot analysis were not significantly different between MEF^PTEN−/−^ and MEF^WT^ (Fig. 5B and inset). Together, these sets of data suggested that the increased expressions of the RCs observed in MEF^PTEN−/−^ were not likely to be dependent on gene transcription.

#### (c) 5′ cap-dependent protein translation

**Figure 6:**
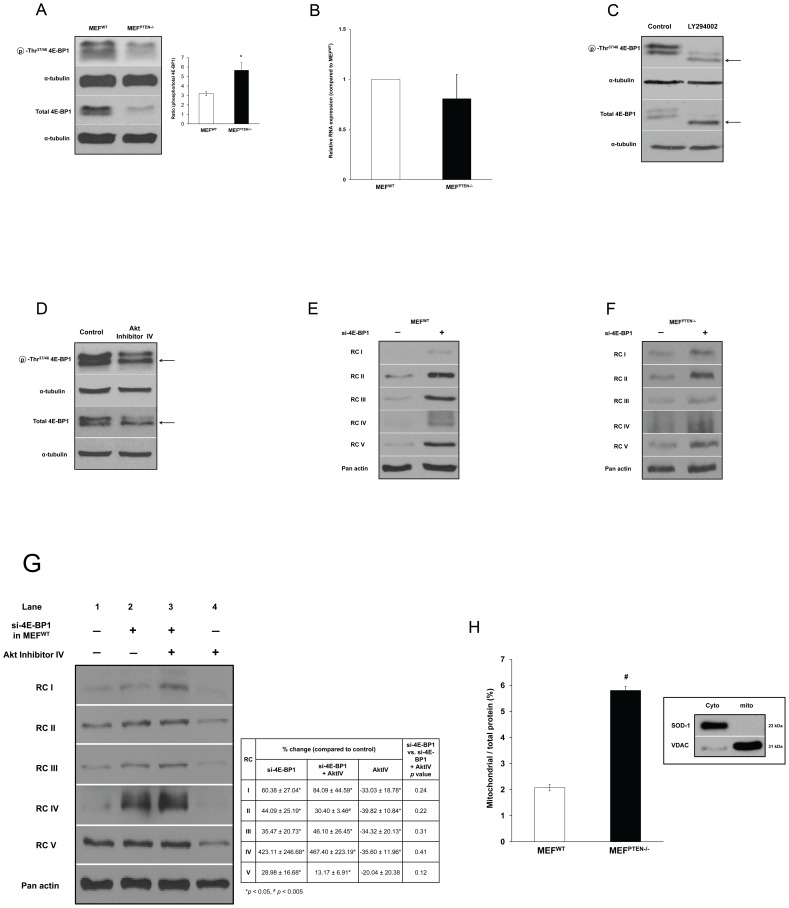
Parameters related to 4E-BP1. (**A**) *Protein expressions of 4E-BP1 and p-Thr^37/46^4E-BP1* MEF^PTEN−/−^ has significantly lower total 4E-BP1 but a higher ratio of phospho- relative to total- 4E-BP1 (inset). * *p*<0.05 for n = 3. (**B**) *mRNA Level of 4E-BP1* The mRNA of 4E-BP1 was not different between MEF^WT^ and MEF^PTEN−/−^ with *p* = 0.231 for n = 3. (**C**) *Effect of LY 294002* Hypo-phosphorylation of 4E-BP1 and p-Thr^37/46^4E-BP1 by LY was observed by downward band shifts in both, as indicated by arrows. (**D**) *Effect of Akt inhibitor IV* Hypo-phosphorylation of p-Thr^37/46^ 4E-BP1 and total 4E-BP1 by Akt inhibitor IV (shown as bands of higher density, indicated by arrows) was observed but no band shift was evident. (**E, F**) *RNA-silencing of 4E-BP1 on protein expressions of RCs in MEF^WT^ and MEF^PTEN−/−^* Up-regulation of the expressions of all RCs was apparent. However, the effect was more pronounced in MEF^WT^ because of its higher expression of 4E-BP1 as shown in Fig. 6A. The fact that RC up-regulation was also observed in MEF^PTEN−/−^ suggested that there was residual 4E-BP1 in the knockout which was indeed shown in Fig. 6A. (**G**) *Gene silencing of 4E-BP1 with and without Akt Inhibitor IV on RC expressions in MEF^WT^* RC expressions were significantly higher with si-4E-BP1 compared with untreated control (Lane 1) in MEF^WT^ in the absence or presence of Akt inhibitor IV as shown in Lanes 2 and 3, respectively (*p<0.05, # p<0.005 relative to untreated controls). Thus, it was evident that in the absence of 4E-BP1, Akt did not affect RC expressions significantly with p values >0.05 (Fig. 6G inset, comparing Lanes 2 and 3). In contrast, in the presence of an Akt inhibitor (AktIV), RC expressions were down-regulated when 4E-BP1 was intact (Lane 4) showing that the action of Akt required 4E-BP1. *p<0.05, # p<0.005 for n = 3. (**H**) *Mitochondrial protein relative to total cellular protein* The ratio was about three times higher in MEF^PTEN−/−^ compared to MEF^WT^. The purity of the mitochondrial preparation was confirmed by the use of SOD-1 (Cu-Zn superoxide dismutase) and VDAC (voltage dependent anion channel), the cytosolic and mitochondrial markers, respectively (inset). ^#^ *p*<0.005 for n = 3.

We next explored if the Akt-mediated mitochondrial function was regulated by protein translation. As mentioned previously, 4E-BP1 has a central role in converging the upstream signals to protein translation [12], [13]. Comparatively, MEF^PTEN−/−^ had lower 4E-BP1 (Fig. 6A) and a higher proportion of p-Thr^37/46^4E-BP1 relative to total 4E-BP1 when compared to MEF^WT^ (Fig. 6A, inset). The mRNA levels of 4E-BP1 in the two cell types were not significantly different (p = 0.148) as shown in Fig. 6B. In addition, the repression of activities and expressions of the RCs by LY294002 and Akt inhibitor IV could also be mediated by 4E-BP1 as incubation with LY294001 resulted in a band shift downwards in total and p-4E-BP1 (shown by arrows in Fig. 6C), a phenomenon which indicated hypo-phosphorylation [11]. Although there was no band shift with Akt inhibitor IV, there was evidence of a more hypo-phosphorylated p-Thr^37/46^ 4E-BP1 and total 4E-BP1 (shown by arrows of lower bands of greater intensity in Fig. 6D). In general, hypo-phosphorylation of 4E-BP1 and/or p-Thr^37/46^ 4E-BP1 would lead to decreased protein translation which was evident in RC I, III and IV expressions in MEF^PTEN−/−^ following inhibition by LY and Akt inhibitor IV (Figs. 4C and 4D). Gene silencing of 4E-BP1 in MEF^WT^ and MEF^PTEN−/−^ up-regulated the expressions of all RCs (Fig. 6E and 6F, respectively). The fact that this was also observed in MEF^PTEN−/−^ suggested that there was residual 4E-BP1 in these cells which was demonstrated in Fig. 6A. The higher degree of increase in RC expressions in the MEF^WT^ by si-4E-BP1 (Fig. 6E) could be explained by their higher basal 4E-BP1 (Fig. 6A). A number of other assorted proteins were also measured following si-4E-BP1, but their protein expressions were not significantly changed, except for Bcl-2 which was down regulated (Figure S1).

#### (d) Direct control of RC expressions by 4E-BP1

To examine if 4E-BP1 exerts control directly downstream of Akt activation, we performed gene silencing on 4E-BP1 in MEF^WT^ followed by inhibition with Akt inhibitor IV. As shown in Fig. 6G, si-RNA of 4E-BP1 up-regulated all the RC expressions in the absence or presence of Akt inhibitor IV (Lanes 2 and 3) as compared to untreated controls (Lane 1), indicated by *p<0.05 and ^#^ *p*<0.005. In other words, with gene silencing of 4E-BP1, the RC expressions were not affected by Akt inhibition as shown by *p*>0.05 in Fig. 6G inset. However, when 4E-BP1 was intact (Lane 4), inhibition of Akt down-regulated RC expressions. This positive control showed the efficiency of the Akt inhibitor IV, which has previously been demonstrated in MEF^PTEN−/−^ (Fig. 4D). Together, the results suggested that regulation of RC expressions by Akt was dependent on 4E-BP1.

#### (e) Increased mitochondrial protein relative to total protein

In view of the mitochondrial location of the RCs, it was conceivable that their up-regulation could contribute to more proteins in this organelle relative to the total cellular protein in MEF^PTEN−/−^. Indeed, we observed a three-fold increase in the ratio of mitochondrial protein relative to total cellular protein in MEF^PTEN−/−^ compared to MEF^WT^ (Fig. 6H). The purity of the mitochondrial preparation was confirmed by the use of SOD-1 and VDAC, the cytosolic and mitochondrial markers respectively (Fig. 6H, inset).

## Discussion

The immortalized MEF^PTEN−/−^ [6] provided a cell model to study any aberrant metabolism resulting from the oncogenic action of Akt. Following its activation, Akt has been reported to translocate to the mitochondria to act on its downstream targets [33]. Thus, there is evidence of a cross-talk between Akt and mitochondria. This study explores if Akt hyperactivation has an impact on mitochondrial metabolism. It is conceivable that accelerated aerobic glycolysis from Akt signaling [14] produces increased pyruvate which is channeled concomitantly to lactate and the TCA cycle. The reducing equivalents, NADH and FADH_2_ generated in the mitochondrial matrix are finally oxidized by the active electron transport system consisting of up-regulated RCs in MEF^PTEN−/−^. These could contribute to the higher MMP and ATP biosynthesis in MEF^PTEN−/−^ and increased oxygen consumption reported in Akt-transfected MEFs [34]. Our study also showed consistently higher net routine oxygen consumption in MEF^PTEN−/−^ which was reproducible by silencing PTEN in the wild type. In both instances, the lower degree of leak respiration may lead to their greater efficiency in OXPHOS. Lower leak is possibly the result of their higher cholesterol content as we had noted consistently that 2–3 times more digitonin was required for cell permeabilization of MEF^PTEN−/−^. It has been reported that cholesterol enhances the rigidness of mitochondrial membranes leading to a lower leak [35]. In addition, higher enzyme activities of the RCs could be another contributing factor. Of particular interest is that the MEF^PTEN−/−^ retain similar relative proportions of activities of RCI, III and IV as those present in the wild type which infers that they could be co-ordinately regulated. These three RCs form supercomplexes known as respirasomes [36], [37] which may subject them to collective regulation. Our ratio of the enzyme activities of 1∶2 for RCIII: RCIV is coincidentally similar to that deduced in the model of the “respiratory string” which is made up of several respirasomes [37].

Two inhibitors of the Akt/mTOR pathway were used to examine if the higher RC activities and expressions were attributed to hyperactivated Akt from PTEN aberration in MEF^PTEN−/−^. They are LY294002 (LY) which targets both PI3K and mTOR [16], [28], [29] and Akt inhibitor IV which has previously been reported to block Akt autokinase activity [30]. Both inhibitors decreased RC enzyme activities and protein expressions of RC I, III, and IV, with little to no change in RCII and RCV, an observation which further supports the concept of organization of these three proton pumping RCs in supercomplexes of respirasomes discussed above [37]. In addition to their co-ordinated regulation, their ease of change could be explained by their different values of ΔG of folding energy and the length of the 5′UTRs [38]. RC IV with a ΔG of −7Kcal/mol and short 5′UTRs could render it more amendable to inhibition and possibly transcription and/or translation in contrast to RCV with a ΔG of −46Kcal/mol which would be more resistant to change as towards LY and Akt inhibitor in this study. Overall, LY seems to exert a greater influence on these reactions as it is an inhibitor of both PI3K and mTOR [16], [28], [29]. Inhibition of mTORC2 by LY would compromise the phosphorylation of Ser^473^ and the subsequent phosphorylation at Thr^308^ of Akt necessary for its full activation [39]. In addition, its inhibition of mTORC1 would have a more direct impact on 4E-BP1 as compared to Akt inhibitor IV which acts upstream. However, the greater effect of LY did not result in a more pronounced inhibition of RC enzyme activities. This may suggest that RC activities are more directly affected by Akt kinase since the Akt inhibitor IV has been reported to compromise the Akt autokinase activity more significantly than wortmanin, another PI3K inhibitor [30]. No attempt is made to correlate the degree of inhibition of enzyme activities with protein expressions of the RCs as Western blot analysis depends on only one of several subunits present in the RC, whereas enzyme activity measures the functionality of the composite RC. A differential mechanism of action of the two inhibitors was also inferred from the distinct band shifts in 4E-BP1 and its phosphorylated form by LY which was not apparent with Akt inhibitor IV.

Although our data of LY and Akt inhibitor IV described above provide a plausible relationship between Akt, 4E-BP1 and RC expressions, siRNA targeting 4E-BP1 is deemed necessary to establish unequivocally a direct association between 4E-BP1 and RC expressions. In eukaryotes, protein synthesis is mainly regulated by 4E-BP1, a repressor of 5′cap-dependent translation [40]. 4E-BP1, a downstream target of mTORC1, binds eIF4E to inhibit protein translation. On phosphorylation, it detaches from eIF4E to initiate protein translation [11]. With gene silencing of 4E-BP1, expressions of RC I to V were up-regulated in MEF^WT^. Likewise, PTEN gene silencing showed a similar effect. The first suggestive evidence of an involvement of translational regulation was the observation of a drastic reduction of this translational repressor in MEF^PTEN−/−^. To examine if up-regulation is peculiar to the RCs, we measured the expressions of assorted proteins (namely cytosolic proteins: ME 1, LDH-V and mitochondrial proteins: HK II, UCP2, ACO2, Bcl-2 and VDAC) besides RCI to V, following silencing of 4E-BP1. Of these, only the RCs were up-regulated which appeared to suggest some degree of specificity of 4E-BP1 regulation on the electron-transport proteins

Generally, the selection of mRNAs for translation is exerted at the initiation stage with binding of elF4F complex to 5′-end of each mRNA and interaction between 3′UTR binding proteins and 4E-BPs [41]. The binding of the mRNA binding proteins of the Pumilio (PUF) family to the 3′ UTR can control mRNA stability and translational repression [42] to provide some measure of selectivity. Of all the Pufs indentified in yeast, Puf3p has been shown to bind nearly exclusively to nuclear encoded mitochondrial mRNAs [43], [44]. Mitochondrial mRNAs, which essentially code for the assembly factors of respiratory chain complexes and the mitochondrial translation machinery are highly dependent on the presence of Puf3 [45]. Taken together, we concluded that it is not impossible for 4E-BP1 to regulate expressions of mitochondrial RCs in a selective manner. However, post-translational modification of enzyme activities cannot be ruled out as several subunits of RCI were predicted from proteomic studies to be potentially capable of phosphorylation, e.g. tyrosine phosphorylation of RCI and RCV [46], [47]. Transcriptional control was deemed unlikely as there was no significant difference in the mRNAs of several subunits of the RCs between MEF^PTEN−/−^ and MEF^WT^. Furthermore, the protein expressions of the transcription factor PGC-1α and its associated downstream targets, NRF-1 and mtTFA were similar between MEF^PTEN−/−^ and MEF^WT^. Our conclusion concurred with the report that the regulation of mitochondrial function by mTOR is independent of transcription [48].

**Figure 7:**
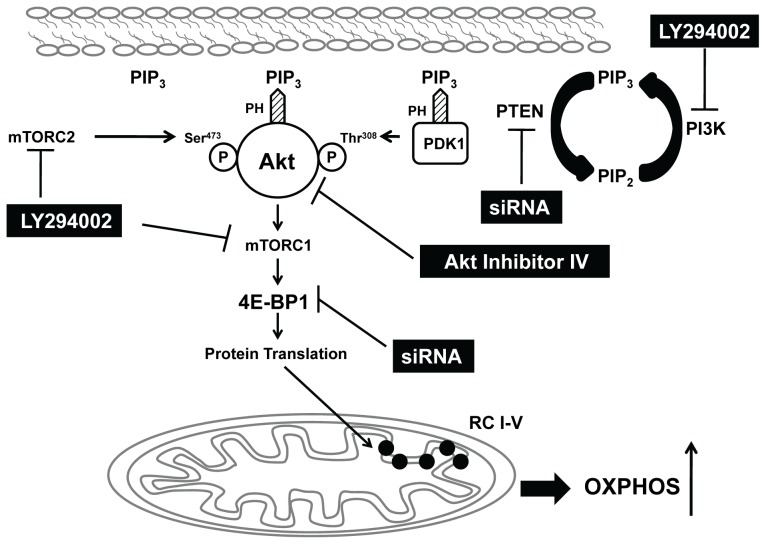
A proposed model of crosstalk between Akt/mTOR/4E-BP1 signaling pathway and mitochondria. PTEN knockout in mouse embryonic fibroblasts (MEF^PTEN−/−^) results in an accumulation of PIP_3_ produced by PI3K at the plasma membrane. The recruitment of Akt and phosphoinositide-dependent protein kinase 1 (PDK1) by PIP_3_ is mediated via their pleckstrin homology (PH) domains. The phosphorylation at Ser^473^ by mTORC2 and that at Thr^308^ by PDK1 leads to a fully activated Akt [39] which then phosphorylates mTORC1. 4E-BP1, the downstream target of mTORC1, on phosphorylation becomes inactive as a repressor of protein translation as it is not able to bind to eIF4E [11]. This leads to the initiation of protein translation of RC components as shown in this study, contributing to enhanced OXPHOS in MEF^PTEN−/−^. Likewise, knockdown of PTEN in MEF^WT^ or of 4E-BP1 in MEF^WT^ and MEF^PTEN−/−^ by RNA silencing also increased RC expressions. Conversely, attenuation of the hyper-activated Akt in MEF^PTEN−/−^ by LY294002 or Akt inhibitor IV leads to lower RC activities and expressions. Together, the data presented supports our hypothesis of a link between Akt/mTOR/4E-BP1 and mitochondrial respiratory function.

In conclusion, the hyperactivated Akt confers on MEF^PTEN−/−^ a bioenergetic advantage by up-regulating their oxidative mitochondrial functions. We propose the enhanced mitochondrial capacity in MEF^PTEN−/−^ is used to support their higher rate of proliferation upon substrate availability. Increased OXPHOS has been shown in H-Ras transformed fibroblast [49] and this bestows a proliferative advantage to K-Ras-mediated tumorigenecity [50] and provides energy to transformed human mesenchymal stem cells [51]. Our hypothesis that mitochondrial respiratory capacity is regulated by the Akt/mTOR/4E-BP1 cascade as summarized in Fig. 7. Supporting evidence was provided using known small molecules as inhibitors of this signaling pathway and siRNA to knockdown PTEN or 4E-BP1 to demonstrate the link between PTEN/Akt/mTOR/4E-BP1 signaling and protein expressions of the electron transport respiratory complexes. Our study has highlighted the role of 4E-BP1 in mitochondrial function in the early-transformed cells with PTEN/Akt aberration.

## Supporting Information

Figure S1 **Gene silencing of 4E-BP1 on other proteins.** There was little to no effect on other proteins examined with siRNA on 4E-BP1 in MEF^WT^ except for Bcl-2 which was decreased, p<0.05. In contrast, there was up-regulation of all respiratory complexes as shown in Figure 6E and 6F.
(TIF)

Table S1 **Primers used in Real-time PCR.** mRNAs of subunits (encoded by mitochondrial and nuclear DNAs) of respiratory complexes were measured using the respective primers as shown. These mRNAs are of the same RC subunits detected by Western blots.
(DOC)

## References

[1] Stokoe D (2001) Pten. Curr Biol 11: R502. 11470419 10.1016/s0960-9822(01)00303-7

[2] Suzuki A, Nakano T, Mak TW, Sasaki T (2008) Portrait of PTEN: messages from mutant mice. Cancer Sci 99: 209–213. 18201277 10.1111/j.1349-7006.2007.00670.x PMC11158684

[3] Li J, Yen C, Liaw D, Podsypanina K, Bose S, et al (1997) PTEN, a putative protein tyrosine phosphatase gene mutated in human brain, breast, and prostate cancer. Science 275: 1943–1947. 9072974 10.1126/science.275.5308.1943

[4] Maehama T, Dixon JE (1998) The tumor suppressor, PTEN/MMAC1, dephosphorylates the lipid second messenger, phosphatidylinositol 3,4,5-trisphosphate. J Biol Chem 273: 13375–13378. 9593664 10.1074/jbc.273.22.13375

[5] Manning BD, Cantley LC (2007) AKT/PKB signaling: navigating downstream. Cell 129: 1261–1274. 17604717 10.1016/j.cell.2007.06.009 PMC2756685

[6] Stambolic V, Suzuki A, de la Pompa JL, Brothers GM, Mirtsos C, et al (1998) Negative regulation of PKB/Akt-dependent cell survival by the tumor suppressor PTEN. Cell 95: 29–39. 9778245 10.1016/s0092-8674(00)81780-8

[7] Hobert JA, Eng C (2009) PTEN hamartoma tumor syndrome: an overview. Genet Med 11: 687–694. 19668082 10.1097/GIM.0b013e3181ac9aea

[8] Blanco-Aparicio C, Renner O, Leal JF, Carnero A (2007) PTEN, more than the AKT pathway. Carcinogenesis 28: 1379–1386. 17341655 10.1093/carcin/bgm052

[9] Xue L, Nolla H, Suzuki A, Mak TW, Winoto A (2008) Normal development is an integral part of tumorigenesis in T cell-specific PTEN-deficient mice. Proc Natl Acad Sci U S A 105: 2022–2027. 18250301 10.1073/pnas.0712059105 PMC2538875

[10] Ma XM, Blenis J (2009) Molecular mechanisms of mTOR-mediated translational control. Nat Rev Mol Cell Biol 10: 307–318. 19339977 10.1038/nrm2672

[11] Gingras AC, Kennedy SG, O'Leary MA, Sonenberg N, Hay N (1998) 4E-BP1, a repressor of mRNA translation, is phosphorylated and inactivated by the Akt(PKB) signaling pathway. Genes Dev 12: 502–513. 9472019 10.1101/gad.12.4.502 PMC316523

[12] Dowling RJ, Topisirovic I, Alain T, Bidinosti M, Fonseca BD, et al (2010) mTORC1-mediated cell proliferation, but not cell growth, controlled by the 4E-BPs. Science 328: 1172–1176. 20508131 10.1126/science.1187532 PMC2893390

[13] Armengol G, Rojo F, Castellvi J, Iglesias C, Cuatrecasas M, et al (2007) 4E-binding protein 1: a key molecular “funnel factor” in human cancer with clinical implications. Cancer Res 67: 7551–7555. 17699757 10.1158/0008-5472.CAN-07-0881

[14] Elstrom RL, Bauer DE, Buzzai M, Karnauskas R, Harris MH, et al (2004) Akt stimulates aerobic glycolysis in cancer cells. Cancer Res 64: 3892–3899. 15172999 10.1158/0008-5472.CAN-03-2904

[15] Pelicano H, Xu RH, Du M, Feng L, Sasaki R, et al (2006) Mitochondrial respiration defects in cancer cells cause activation of Akt survival pathway through a redox-mediated mechanism. J Cell Biol 175: 913–923. 17158952 10.1083/jcb.200512100 PMC2064701

[16] Brunn GJ, Williams J, Sabers C, Wiederrecht G, Lawrence JC Jr, et al (1996) Direct inhibition of the signaling functions of the mammalian target of rapamycin by the phosphoinositide 3-kinase inhibitors, wortmannin and LY294002. EMBO J 15: 5256–5267. 8895571 PMC452270

[17] Pesta D, Gnaiger E (2012) High-resolution respirometry: OXPHOS protocols for human cells and permeabilized fibers from small biopsies of human muscle. Methods Mol Biol 810: 25–58. 22057559 10.1007/978-1-61779-382-0_3

[18] Pallotti F, Lenaz G (2001) Isolation and subfractionation of mitochondria from animal cells and tissue culture lines. Methods Cell Biol 65: 1–35. 11381588 10.1016/s0091-679x(01)65002-7

[19] Zhang X, Vincent AS, Halliwell B, Wong KP (2004) A mechanism of sulfite neurotoxicity: direct inhibition of glutamate dehydrogenase. J Biol Chem 279: 43035–43045. 15273247 10.1074/jbc.M402759200

[20] Ng LE, Vincent AS, Halliwell B, Wong KP (2006) Action of diclofenac on kidney mitochondria and cells. Biochem Biophys Res Commun 348: 494–500. 16890207 10.1016/j.bbrc.2006.07.089

[21] Lim HY, Ho QS, Low J, Choolani M, Wong KP (2011) Respiratory competent mitochondria in human ovarian and peritoneal cancer. Mitochondrion 11: 437–443. 21211574 10.1016/j.mito.2010.12.015

[22] Birch-Machin MA, Briggs HL, Saborido AA, Bindoff LA, Turnbull DM (1994) An evaluation of the measurement of the activities of complexes I–IV in the respiratory chain of human skeletal muscle mitochondria. Biochem Med Metab Biol 51: 35–42. 8192914 10.1006/bmmb.1994.1004

[23] Ragan CI, Wilson, M T., Darley-Usmar V.M., Lowe P.N., (1987) Chapter 4: Subfractionation of mitochondria and isolation of the proteins of oxidative phosporylation. In Mitochondria, a practical approach (Darley-Usmar, VM, Rickwood, D, Wilson, MT, ed): pp 79–112.

[24] Trounce IA, Kim YL, Jun AS, Wallace DC (1996) Assessment of mitochondrial oxidative phosphorylation in patient muscle biopsies, lymphoblasts, and transmitochondrial cell lines. Methods Enzymol 264: 484–509. 8965721 10.1016/s0076-6879(96)64044-0

[25] Barrientos A (2002) In vivo and in organello assessment of OXPHOS activities. Methods 26: 307–316. 12054921 10.1016/S1046-2023(02)00036-1

[26] Lim HW, Lim HY, Wong KP (2009) Uncoupling of oxidative phosphorylation by curcumin: implication of its cellular mechanism of action. Biochem Biophys Res Commun 389: 187–192. 19715674 10.1016/j.bbrc.2009.08.121

[27] Smith PK, Krohn RI, Hermanson GT, Mallia AK, Gartner FH, et al (1985) Measurement of protein using bicinchoninic acid. Anal Biochem 150: 76–85. 3843705 10.1016/0003-2697(85)90442-7

[28] Feldman ME, Apsel B, Uotila A, Loewith R, Knight ZA, et al (2009) Active-site inhibitors of mTOR target rapamycin-resistant outputs of mTORC1 and mTORC2. PLoS Biol 7: 0371–0383. 10.1371/journal.pbio.1000038 PMC2637922 19209957

[29] Thoreen CC, Kang SA, Chang JW, Liu Q, Zhang J, et al (2009) An ATP-competitive mammalian target of rapamycin inhibitor reveals rapamycin-resistant functions of mTORC1. J Biol Chem 284: 8023–8032. 19150980 10.1074/jbc.M900301200 PMC2658096

[30] Pastorino JG, Hoek JB, Shulga N (2005) Activation of glycogen synthase kinase 3beta disrupts the binding of hexokinase II to mitochondria by phosphorylating voltage-dependent anion channel and potentiates chemotherapy-induced cytotoxicity. Cancer Res 65: 10545–10554. 16288047 10.1158/0008-5472.CAN-05-1925

[31] Kelly DP, Scarpulla RC (2004) Transcriptional regulatory circuits controlling mitochondrial biogenesis and function. Genes Dev 18: 357–368. 15004004 10.1101/gad.1177604

[32] Scarpulla RC (2011) Metabolic control of mitochondrial biogenesis through the PGC-1 family regulatory network. Biochim Biophys Acta 1813: 1269–1278. 20933024 10.1016/j.bbamcr.2010.09.019 PMC3035754

[33] Bijur GN, Jope RS (2003) Rapid accumulation of Akt in mitochondria following phosphatidylinositol 3-kinase activation. J Neurochem 87: 1427–1435. 14713298 10.1046/j.1471-4159.2003.02113.x PMC2040497

[34] Nogueira V, Park Y, Chen CC, Xu PZ, Chen ML, et al (2008) Akt determines replicative senescence and oxidative or oncogenic premature senescence and sensitizes cells to oxidative apoptosis. Cancer Cell 14: 458–470. 19061837 10.1016/j.ccr.2008.11.003 PMC3038665

[35] Baggetto LG, Clottes E, Vial C (1992) Low mitochondrial proton leak due to high membrane cholesterol content and cytosolic creatine kinase as two features of the deviant bioenergetics of Ehrlich and AS30-D tumor cells. Cancer Res 52: 4935–4941. 1516050

[36] Dudkina NV, Kudryashev M, Stahlberg H, Boekema EJ (2011) Interaction of complexes I, III, and IV within the bovine respirasome by single particle cryoelectron tomography. Proc Natl Acad Sci U S A 108: 15196–15200. 21876144 10.1073/pnas.1107819108 PMC3174662

[37] Wittig I, Carrozzo R, Santorelli FM, Schagger H (2006) Supercomplexes and subcomplexes of mitochondrial oxidative phosphorylation. Biochim Biophys Acta 1757: 1066–1072. 16782043 10.1016/j.bbabio.2006.05.006

[38] Zid BM, Rogers AN, Katewa SD, Vargas MA, Kolipinski MC, et al (2009) 4E-BP extends lifespan upon dietary restriction by enhancing mitochondrial activity in Drosophila. Cell 139: 149–160. 19804760 10.1016/j.cell.2009.07.034 PMC2759400

[39] Sarbassov DD, Guertin DA, Ali SM, Sabatini DM (2005) Phosphorylation and regulation of Akt/PKB by the rictor-mTOR complex. Science 307: 1098–1101. 15718470 10.1126/science.1106148

[40] Sonenberg N (1996) Chapter 8: mRNA 5′ Cap-binding Protein eIF4E and Control of Cell Growth. In Translational Control (Hershey, John W B,Mathews, Michael,Sonenberg, Nahum, ed) Cold Spring Harbor Laboratory Press NY: pp. 245–269.

[41] Cridge AG, Castelli LM, Smirnova JB, Selley JN, Rowe W, et al (2010) Identifying eIF4E-binding protein translationally-controlled transcripts reveals links to mRNAs bound by specific PUF proteins. Nucleic Acids Res 38: 8039–8050. 20705650 10.1093/nar/gkq686 PMC3001062

[42] Wickens M, Bernstein DS, Kimble J, Parker R (2002) A PUF family portrait: 3′UTR regulation as a way of life. Trends Genet 18: 150–157. 11858839 10.1016/s0168-9525(01)02616-6

[43] Olivas W, Parker R (2000) The Puf3 protein is a transcript-specific regulator of mRNA degradation in yeast. EMBO J 19: 6602–6611. 11101532 10.1093/emboj/19.23.6602 PMC305854

[44] Gerber AP, Herschlag D, Brown PO (2004) Extensive association of functionally and cytotopically related mRNAs with Puf family RNA-binding proteins in yeast. PLoS Biol 2: E79. 15024427 10.1371/journal.pbio.0020079 PMC368173

[45] Saint-Georges Y, Garcia M, Delaveau T, Jourdren L, Le Crom S, et al (2008) Yeast mitochondrial biogenesis: a role for the PUF RNA-binding protein Puf3p in mRNA localization. PLoS One 3: e2293. 18523582 10.1371/journal.pone.0002293 PMC2387061

[46] Augereau O, Claverol S, Boudes N, Basurko MJ, Bonneu M, et al (2005) Identification of tyrosine-phosphorylated proteins of the mitochondrial oxidative phosphorylation machinery. Cell Mol Life Sci 62: 1478–1488. 15924266 10.1007/s00018-005-5005-7 PMC11139224

[47] Koopman WJ, Nijtmans LG, Dieteren CE, Roestenberg P, Valsecchi F, et al (2010) Mammalian mitochondrial complex I: biogenesis, regulation, and reactive oxygen species generation. Antioxid Redox Signal 12: 1431–1470. 19803744 10.1089/ars.2009.2743

[48] Ramanathan A, Schreiber SL (2009) Direct control of mitochondrial function by mTOR. Proc Natl Acad Sci U S A 106: 22229–22232. 20080789 10.1073/pnas.0912074106 PMC2796909

[49] de Groof AJ, te Lindert MM, van Dommelen MM, Wu M, Willemse M, et al (2009) Increased OXPHOS activity precedes rise in glycolytic rate in H-RasV12/E1A transformed fibroblasts that develop a Warburg phenotype. Mol Cancer 8: 54. 19646236 10.1186/1476-4598-8-54 PMC2734543

[50] Weinberg F, Hamanaka R, Wheaton WW, Weinberg S, Joseph J, et al (2010) Mitochondrial metabolism and ROS generation are essential for Kras-mediated tumorigenicity. Proc Natl Acad Sci U S A 107: 8788–8793. 20421486 10.1073/pnas.1003428107 PMC2889315

[51] Funes JM, Quintero M, Henderson S, Martinez D, Qureshi U, et al (2007) Transformation of human mesenchymal stem cells increases their dependency on oxidative phosphorylation for energy production. Proc Natl Acad Sci U S A 104: 6223–6228. 17384149 10.1073/pnas.0700690104 PMC1851087

