# Supplementary figures and images for: PTEN/Akt Signaling Controls Mitochondrial Respiratory Capacity through 4E-BP1
Source: PLoS One. 2012 Sep 26;7(9):e45806. doi: 10.1371/journal.pone.0045806 (PMC3458951; doi:10.1371/journal.pone.0045806)

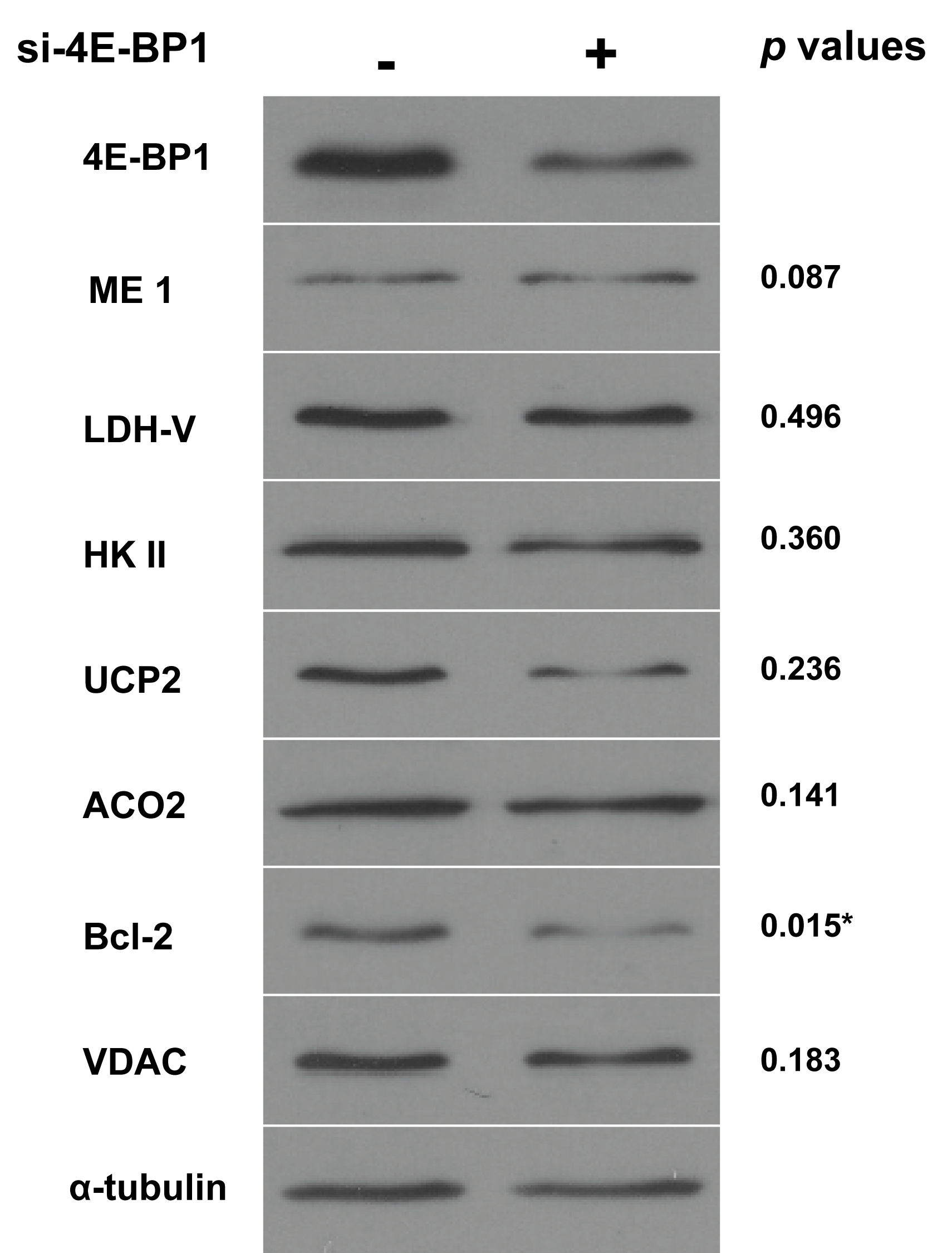

Supplement: Figure S1 — Gene silencing of 4E-BP1 on other proteins. There was little to no effect on other proteins examined with siRNA on 4E-BP1 in MEFWT except for Bcl-2 which was decreased, p<0.05. In contrast, there was up-regulation of all respiratory complexes as shown in Figure 6E and 6F. (TIF) [file pone.0045806.s001.tif]
