# Supplementary material for: PTEN/Akt Signaling Controls Mitochondrial Respiratory Capacity through 4E-BP1
Source: PLoS One. 2012 Sep 26;7(9):e45806. doi: 10.1371/journal.pone.0045806 (PMC3458951; doi:10.1371/journal.pone.0045806)
Supplement: Table S1 — Primers used in Real-time PCR. mRNAs of subunits (encoded by mitochondrial and nuclear DNAs) of respiratory complexes were measured using the respective primers as shown. These mRNAs are of the same RC subunits detected by Western blots. (DOC) [file pone.0045806.s002.doc]

**Table S1** **Primers used in Real-time PCR** mRNAs of subunits (encoded by mitochondrial and nuclear DNAs) of respiratory complexes were measured using the respective primers as shown. These mRNAs are of the same RC subunits detected by Western blots.

| **Encoded by** | **Gene** | **NCBI Reference Sequence** | **Forward (5’-3’)** | **Reverse (5’-3’)** |
| --- | --- | --- | --- | --- |
| **Nuclear**  **DNA** | NDU FB8 | NM_026061 | CGTGTTCCCTTCCTACCA | CCATCAAGCCTCCTCAGA |
|  | SdhB | NM_023374 | CCAGAGACGACTTCACAGA | GCAGCGGTAGACAGAGA |
|  | RC III core 2 | NM_025899 | ATTGAAGCAGTTGGTGGTA | GGTGACATTGAGCAGGAA |
|  | ATP5 F1α1 | NM_007505 | TTAGAGACAACGGCAAGC | CGGAGCAACAGAGACATC |
| **Mitochondrial DNA** | CO1 | NC_005089, GeneID:[17708](http://www.ncbi.nlm.nih.gov/sites/entrez?db=gene&cmd=Retrieve&dopt=full_report&list_uids=17708) | TTCTCCTTCTCCTAGCATCAT | GGTAGACTGTTCATCCTGTTC |
